# Cutaneous Tuberculosis Presenting as a Chronic Dermatologic Disorder

**DOI:** 10.1155/crdm/3172006

**Published:** 2026-02-11

**Authors:** Olivia Spina, Henry Fraimow, Justin Green

**Affiliations:** ^1^ Cooper Medical School of Rowan University, Camden, New Jersey, USA, rowan.edu; ^2^ Department of Infectious Disease, Cooper University Hospital, Camden, New Jersey, USA, cooperhealth.org; ^3^ Division of Dermatology, Cooper University Hospital, Camden, New Jersey, USA, cooperhealth.org

**Keywords:** antituberculous therapy, case report, cutaneous tuberculosis, diagnosis

## Abstract

Cutaneous tuberculosis (CTB) is a rare manifestation of extrapulmonary tuberculosis that is frequently misdiagnosed due to its diverse clinical presentation and resemblance to other dermatological conditions. Tuberculosis verrucosa cutis (TBVC), one clinical manifestation of CTB, poses a particular diagnostic challenge, as lesions are often paucibacillary, resulting in negative culture and PCR results. We present the case of a 73‐year‐old woman with a 10‐year history of recurrent skin lesions on her left hand, initially diagnosed as eczema and exacerbated by topical corticosteroid treatment. Despite repeated negative histopathological stains, mycobacterial cultures. and PCR for *Mycobacterium tuberculosis,* a strong positive QuantiFERON‐TB Gold test established the diagnosis. This case emphasizes the diagnostic utility of interferon‐gamma release assays (IGRAs) in paucibacillary forms of CTB. Management was complicated by adverse drug reactions (ADRs) to first‐line antituberculosis therapy (HRZE), including myalgias, fatigue, and a pruritic rash attributed to pyrazinamide and rifampin. These agents were discontinued, and the patient was transitioned to an alternative regimen, which resulted in improved tolerability and marked clinical improvement. This case highlights the diagnostic pitfalls and therapeutic challenges in managing TBVC. It underscores the importance of maintaining a high index of clinical suspicion for CTB in chronic, verrucous skin lesions, even in patients without specific TB risk factors. It also emphasizes the need for individualized treatment strategies.

## 1. Introduction

Tuberculosis (TB) remains a significant public health concern. While pulmonary involvement is the most common presentation, extrapulmonary TB accounts for between 8% and 24% of all cases [1]. Cutaneous tuberculosis (CTB), an uncommon manifestation of extrapulmonary TB, represents 1.5%–3% of these extrapulmonary cases [1]. Due to its rarity and morphological diversity, CTB is often misdiagnosed, particularly when it mimics other dermatological conditions [1, 2]. Dermatologists, therefore, play a crucial role in early identification and management.

The primary causative agent of CTB is *M. tuberculosis* (MTB) [1, 2]. CTB that presents via the inoculation route of infection in individuals without previous exposure presents as TB chancre, lupus vulgaris, scrofuloderma, or tuberculosis verrucosa cutis (TBVC). Clinical presentation is impacted by the route of infection and host immunity [1, 2]. Multibacillary forms, such as scrofuloderma, possess high bacillary loads and are often easier to confirm microbiologically. In contrast, paucibacillary types, such as TBVC and lupus vulgaris, commonly result in negative staining and cultures, often due to a low bacillary load in the lesion [3, 4].

CTB diagnosis requires a high index of clinical suspicion due to its variable presentation and diagnostic limitations. Although cultures are the gold standard for diagnosis, sensitivity is low (0%–62.8%) and results can take up to 8 weeks. Early lesions may present as suppurative reactions of the epidermis with neutrophils, lymphocytes, and plasma cells densely infiltrating the dermis, accompanied by the classic finding of tuberculoid granulomas [4]. Varying presentations of specific CTB types are detailed in Table 1. However, up to 30% of CTB biopsies fail to show characteristic histology or positive acid‐fast bacilli (AFB) stain [1, 3, 4]. These factors collectively contribute to significant diagnostic delays. Thus, diagnosis often requires integrating clinical, histological, and immunological data.

**TABLE 1:** Classifications of true cutaneous tuberculosis [5–7].

Type	Mode of infection	Presentation	Histology	AFB stain status	Immune status
Tuberculous chancre	Inoculation	Red/brown papules that progress to painless ulcerations with undermined borders	Suppurative granulomatous infiltrate that may present with dermal necrosis	Positive	No prior TB exposure
TB verrucosa cutis	Inoculation	Verrucous plaques commonly on lower legs and feet	Pseudoepitheliomatous hyperplasia, hyperkeratosis, tuberculoid granulomas, and neutrophilic microabscesses in the papillary dermis	Negative	Good immunity
Lupus vulgaris	Hematogenous lymphatic, or contiguous	Multiple red/brown plaques with central atrophy commonly on the head, neck, arms, or legs	Tuberculoid granulomas with minimal caseation, epidermal changes vary	Negative	Moderate to low immunity
Acute miliary TB	Hematogenous	Widespread red papules and pustules of varying size on the trunk or extremities	Poorly formed granulomas with abundant mycobacteria and possible necrosis	Positive	Severely high immunity
TB gumma	Hematogenous	Subcutaneous ulcerating nodules that develop sinus tracts	Diffuse granulomatous inflammation and caseation in the reticular dermis	Positive	Low immunity
Scrofuloderma	Contiguous	Violaceous, ulcerating plaques commonly overlying lymph nodes	Tuberculoid granulomas with caseation and neutrophilic abscesses	Positive	Low immunity
Orifical TB	Autoinoculation	Painful, nonhealing oral ulcers	Epidermal changes vary from ulceration to acanthosis, caseating granulomas commonly present	Positive	Low immunity

Management with standard anti‐TB therapy of isoniazid, rifampin, and pyrazinamide daily plus pyridoxine (HRZE) is effective but often complicated by adverse drug reactions (ADRs), such as those detailed in Table 2. Cutaneous reactions, ranging from mild exanthems to life‐threatening Stevens–Johnson syndrome, are among the most common ADRs and typically appear in the first few weeks of therapy [6, 8]. Additional ADRs such as hepatotoxicity, myalgia, and arthralgias are linked to pyrazinamide therapy [6, 9].

**TABLE 2:** Commonly reported adverse effects of common anti‐TB therapies [8].

Common adverse effect	Suspected agent
Flushing reactions	Rifampicin and pyrazinamide
Urticaria	Any drug
Nausea and vomiting	Isoniazid, ethambutol, pyrazinamide, rifampin
Gastritis and abdominal pain	Ethambutol, fluoroquinolone, isoniazid and pyrazinamide
Hepatitis	Rifampin, pyrazinamide, isoniazid
Arthralgia	Pyrazinamide, fluoroquinolones
Tendonitis and tendon rupture	Fluoroquinolones
Tinnitus and dizziness	Isoniazid, fluoroquinolones
Seizures and peripheral neuropathy	Isoniazid, fluoroquinolones
Psych issues (depression, suicidal ideation, general psychotic symptoms)	Isoniazid, fluoroquinolones
QT prolongation	Fluoroquinolones

This case report highlights the diagnostic complexity of CTB and the therapeutic challenges created by drug intolerance. It emphasizes the role of interferon‐gamma release assays (IGRAs) in diagnosis and the importance of closely monitoring patient‐specific modifications to HRZE.

## 2. Case Report

A 73‐year‐old woman, born in Japan and living in the United States since age two, presented to the dermatology clinic with a 10‐year history of recurrent skin lesions on her left hand. Her medical history included prediabetes, hyperlipidemia, and hypertension. She denied any systemic symptoms, history of TB infection or disease, contact with TB, or recent travel. A tuberculin skin test performed approximately 50 years ago was negative, and she had not received subsequent skin tests since.

**FIGURE 1:**
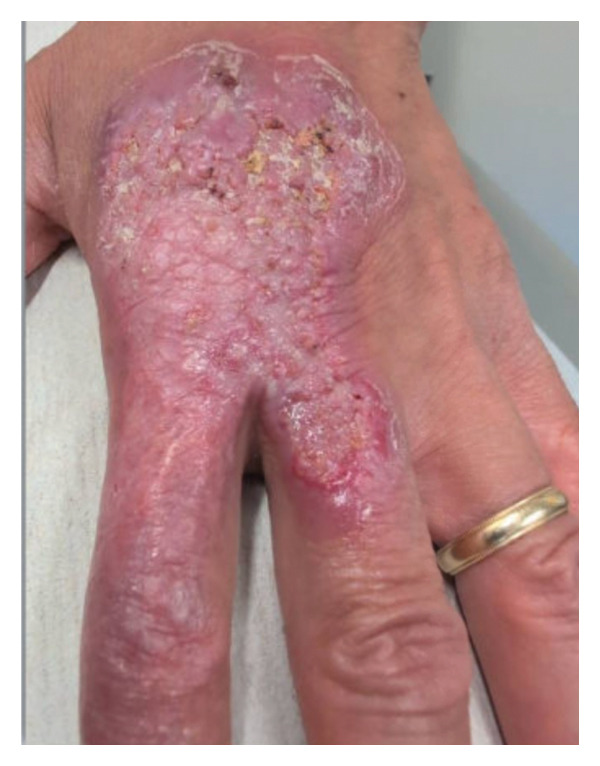
Lesion on the second and third left hand digits extends up the dorsal surface of the left hand observed at first encounter.

The skin lesion developed following a minor injury to her left index finger from a blood lancet. The left index finger initially swelled at the distal aspect, specifically the distal interphalangeal joint, with minor pain that spontaneously resolved. Over time, the patient experienced multiple instances of swelling on the same finger with small, spontaneously resolving papules and nodules. The lesion eventually evolved into a chronic, scaling, and progressive indurated eruption with serous drainage on the dorsal surface of the second and third digits, extending up the hand over 1 year (Figure 1). This progression prompted her to seek medical care.

She was initially diagnosed with eczematous dermatitis and treated with topical corticosteroids, which exacerbated the lesion. This failure prompted a revised differential diagnosis that included an infectious process or deep fungal infection. A skin biopsy demonstrated suppurative granulomatous dermatitis; however, AFB, fungal, and periodic acid–Schiff (PAS) stains were negative. The biopsy raised clinical suspicion for CTB, and dermatology ordered a QuantiFERON‐TB Gold test. The test returned strongly positive (TB1 and TB2 antigens > 5 IU/mL). Given the high clinical suspicion and positive diagnostic results for TB, she was referred to the infectious disease (ID) clinic for further management.

Physical examination at the first ID clinic encounter revealed oozing on the distal third digit and dorsal hand induration (Figure 1). Cultures and PCR for MTB, bacteria, and fungi were all negative. Given the strongly positive IGRA test and the chronicity of the lesion, TBVC was considered the most likely diagnosis. Non‐TB mycobacteria infections, such as *M. marinum and M. kansasii*, were considered but deemed less likely.

**FIGURE 2:**
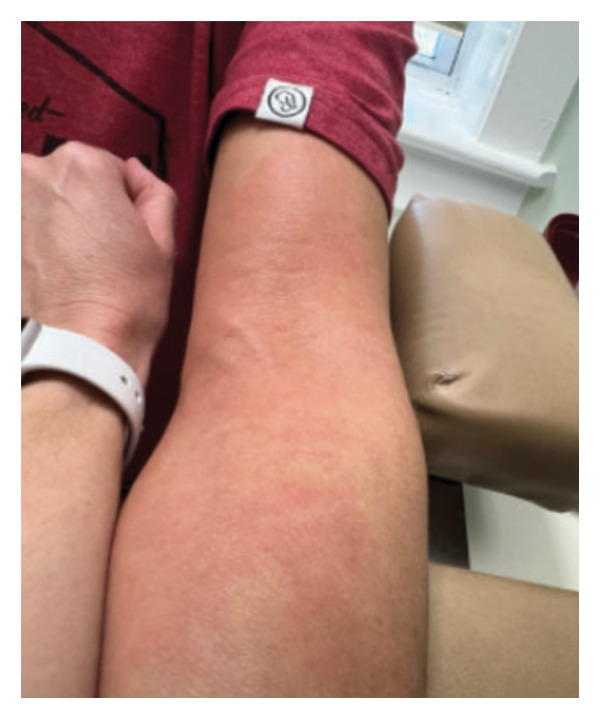
Mild pruritic rash that developed as an adverse reaction to the initial ATT regimen.

**FIGURE 3:**
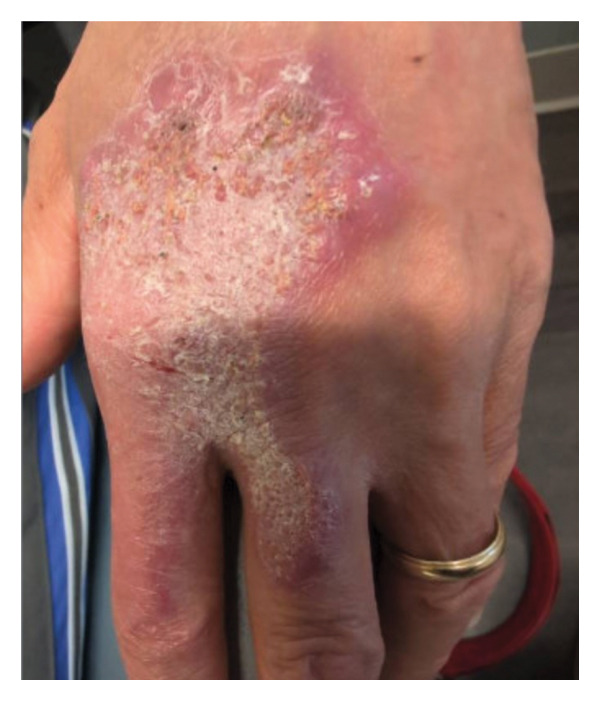
Lesion at the second patient encounter, following regimen changes. The lesion appeared to have slightly improved with noted scaliness.

Empiric HRZE (isoniazid, rifampin, and pyrazinamide daily plus pyridoxine) was initiated. Directly observed therapy for HRZE was provided by her local health department. Ten days into treatment, the patient developed myalgia, fatigue, anorexia with 4‐pound weight loss, and morbilliform exanthema (Figure 2). Pyrazinamide was suspected and discontinued. However, the drug rash worsened and became severely pruritic, prompting cessation of all HRZE medications. The patient was eager to resume therapy, as the original lesion showed improvement, despite ADRs (Figure 3).

After symptomatic management with loratadine and a brief cessation of all therapy, she successfully resumed isoniazid and pyridoxine without adverse effects. However, after she was rechallenged with rifampin, she developed severe myalgias without rash, and rifampin was permanently discontinued. The patient was then transitioned to a new regimen of ethambutol, isoniazid, levofloxacin, plus pyridoxine. She tolerated this new regimen without significant toxicity. After approximately 1 month of the new regimen, the lesion showed progressive improvement with marked flattening and resolution of fissures.

**FIGURE 4:**
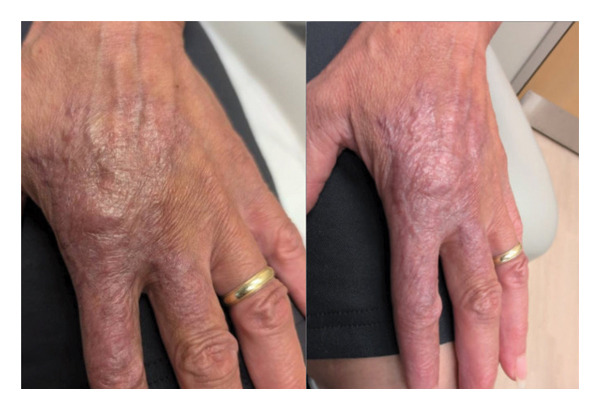
Lesion appearance on the fifth visit after ∼7 months of treatment. Significant improvement is noted.

After 7 months of follow‐up, the patient continued to tolerate her adjusted regimen. Her skin lesion resolved dramatically (Figure 4). The patient completed 8 months of therapy. Given the localized nature and low bacillary burden of the TBVC lesion, this duration was considered sufficient despite the absence of rifampin in her regimen. She continued the regimen for approximately 8 months. She is currently no longer on the regimen and scheduled for a treatment completion visit.

## 3. Discussion

CTB is a rare and often overlooked form of extrapulmonary TB [1]. The diverse clinical morphologies and diagnostic challenges posed by paucibacillary forms lead to significant delays in diagnosis and potential misdiagnoses [1, 3]. This case highlights the complexities inherent in diagnosing and managing CTB, particularly given its protracted course, initial atypical presentation, and the subsequent challenges presented by ADR to the standard anti‐TB treatment.

The patient’s lesion, which followed minor trauma and progressed over a decade into a verrucous, scaling plaque with drainage, is classic for TBVC [1–3]. TBVC is classified as a “true cutaneous TB” and occurs due to direct inoculation of the skin with MTB [1, 2]. The characteristic verrucous, slow‐growing plaque often leads to delayed recognition and treatment, as observed in this case [1–3]. TBVC accounts for just 3%–19% of all CTB cases, making it a rare diagnosis [1].

The clinical presentation in this case, such as slow progression and initial misdiagnosis, is consistent with other published reports of TBVC. Similar published cases have been described as also involving progressive verrucous plaques with chronic persistence. These lesions were often refractory to initial topical or oral therapies, contributing to delayed diagnosis [10–12]. The TBVC lesions in this case were initially diagnosed as eczema. However, like other cases, they were refractory to initial treatment involving corticosteroids [10–12]. Notably, most documented cases involved the lower extremities, differentiating them from this patient’s upper extremity involvement [10–12].

Microbiological confirmation of TBVC is notoriously difficult. Negative microbiological and histological findings are characteristic of TBVC due to sparse amounts of mycobacteria in the lesion, and even molecular testing for MTB DNA may be negative [5, 6]. The patient did not receive any tuberculin skin testing; however, the histological findings of the lesion prompted a QuantiFERON‐TB Gold test that returned strongly positive, guiding appropriate clinical suspicion and initiation of treatment. Histopathology was only provided as a summary report and therefore could be considered a limitation due to the inability to see the histopathological slides. This highlights the crucial use of IGRA testing to diagnose challenging cases of TB, especially paucibacillary forms where direct detection of mycobacteria may be difficult. IGRA testing has been helpful in the diagnosis of other TBVC cases [11].

However, the utility of IGRAs has limitations. IGRAs rely on cell‐mediated immunity and, like tuberculin skin tests, cannot accurately distinguish between latent and active TB infections [13]. Further, systematic reviews have concluded that active TB can neither be ruled in nor out with IGRAs due to their suboptimal sensitivity for active TB diagnosis [10]. Thus, IGRA testing alone cannot confirm a diagnosis of TB.

This case also illustrated the therapeutic complexity of CTB, particularly in patients with intolerance to first‐line agents. Adverse reactions to rifampin, pyrazinamide, and isoniazid are frequently observed [1–3, 8]. This patient experienced myalgia, fatigue, anorexia, and pruritic morbilliform exanthema that led to discontinuation of treatment. The morbilliform exanthema falls under the umbrella of cutaneous adverse drug reactions (CADRs). CADR may be confined to the skin or involved in a multisystem reaction [1, 2, 7]. They typically occur during the first few weeks of therapy and may resolve on their own [7, 14].

Evidence suggests that pyrazinamide is the most frequent offender of CADR, and thus pyrazinamide must be of high suspicion for CADRs [14]. Rechallenge is still essential, however, as it was in this case. Guidelines recommend rechallenge after complete resolution of the CADR in 2‐3‐day intervals starting with rifampin, followed by isoniazid, then ethambutol or pyrazinamide [7, 14]. Due to the recurrence of ADRs with rifampin rechallenge, it was removed from the patient’s regimen. While rifampin‐based regimens are considered superior for TB outcomes, this case illustrated a scenario where intolerance necessitated an alternative approach [2, 3].

This case provides several important implications for clinical practice. Clinicians should maintain a high index of suspicion for CTB in the differential diagnosis of chronic, treatment‐resistant skin lesions. IGRA testing can be crucial in guiding the diagnosis toward CTB, particularly in paucibacillary forms of CTB. Rapid improvement following the initiation of HRZE provides confirmation of the diagnosis. In addition, ADRs to HRZE are common and require flexible, patient‐centered management to ensure successful treatment completion.

## 4. Conclusion

TBVC is a rare but challenging diagnosis among the varieties of extrapulmonary TB. This case underscores the diagnostic challenge posed by its chronic, indolent nature and ability to mimic common dermatological conditions. The diagnostic process was further complicated by the paucibacillary nature of the lesions, which often yields negative results on many histological and microbiological findings. Despite these limitations, clinical suspicion and a positive IGRA were instrumental in confirming the diagnosis.

Management was further challenged by significant intolerance to pyrazinamide and rifampin, necessitating several regimen changes to establish a tolerable and effective treatment plan. This patient’s experience highlights the importance of close monitoring for ADRs in patients receiving HRZE and the necessity of flexible treatment plans to balance efficacy and tolerability. Early recognition, appropriate testing, and tailored therapy are essential to improving outcomes for patients with CTB.

## Author Contributions

Olivia Spina: main writer and editor of the case report.

Henry Fraimow, MD: corresponding author who provided direct infectious disease care for the patient and collected the necessary information and data.

Justin Green, MD: corresponding author who provided direct dermatological care for the patient and collected the necessary information and data.

## Funding

The authors declare that they received no external funding for this work.

## Consent

Written informed consent was obtained from the patient for participation and publication of all relevant clinical information and images.

## Conflicts of Interest

The authors declare no conflicts of interest.

## Data Availability

The data that support the findings of this study are available upon request from the corresponding author. The data are not publicly available due to privacy or ethical restrictions.
